# Mind the Gap: Recurrence of Sex-Related Differences in Patients with Acute Atrial Fibrillation in the Emergency Department—A Retrospective Cohort Study

**DOI:** 10.3390/jcm14041250

**Published:** 2025-02-13

**Authors:** Sophie Gupta, Martin Lutnik, Filippo Cacioppo, Julia Oppenauer, Teresa Lindmayr, Nikola Schütz, Elvis Tumnitz, Hans Domanovits, Michael Schwameis, Jan Niederdöckl

**Affiliations:** 1 Department of Emergency Medicine, Medical University of Vienna, 1090 Vienna, Austria; sophie.gupta@meduniwien.ac.at (S.G.); filippo.cacioppo@meduniwien.ac.at (F.C.); julia.oppenauer@meduniwien.ac.at (J.O.); teresa.lindmayr@meduniwien.ac.at (T.L.); nikola.schuetz@meduniwien.ac.at (N.S.); elvis.tumnitz@meduniwien.ac.at (E.T.); hans.domanovits@meduniwien.ac.at (H.D.); jan.niederdoeckl@meduniwien.ac.at (J.N.); 2 Department of Clinical Pharmacology, Medical University of Vienna, 1090 Vienna, Austria; martin.lutnik@meduniwien.ac.at

**Keywords:** atrial fibrillation/flutter, arrhythmia, cardioversion, sex-specific treatment, sex gap, emergency medicine

## Abstract

**Background/Objectives**: In recent years, awareness of sex disparities in atrial fibrillation (AF) and atrial flutter (AFL) has grown, resulting in significant advancements in sex-specific treatment strategies. As these treatment approaches continue to evolve, it is essential to remain attentive to sex-related issues to ensure equitable care for all patients, a point first emphasised by the 2016 AF guidelines. Our objective was the long-term evaluation of sex-specific treatment standards for acute AF/AFL. **Methods**: This cohort study included cases of acute AF/AFL treated in the emergency department of the Medical University of Vienna, Austria, between 2012 and 2022. The Kaplan–Meier method was used to analyse time-to-event data. The effect of sex on the time to restoration of sinus rhythm was assessed using the log-rank test for unadjusted models and the likelihood ratio test for adjusted models. The groups were categorised based on cases occurring before and after 2016. **Results**: A total of 3661 cases (55.7% male) were analysed. Before 2016, sinus rhythm was achieved in 70.8% of males and 71.2% of females; after 2016, these rates were 71.8% and 68.6%, respectively. The adjusted model showed a significant effect of sex on the time to restoration of sinus rhythm after 2016 (*p* = 0.013) but not before (*p* = 0.865). A subgroup analysis indicated similar results for pharmacologic rhythm control (*p* = 0.035 vs. *p* = 0.193). A sensitivity analysis confirmed robustness, with similar effects in both models. The success rate of pharmacological cardioversion was lower in women than in men but was used more frequently in women after 2016. **Conclusions**: The re-emergence of a sex gap in success rates and time to restoration of sinus rhythm in emergency treatment for acute AF/AFL is concerning and necessitates a re-evaluation of treatment protocols, improved decision-making processes, and further research to ensure equitable, effective, and safe emergency care for all patients.

## 1. Background

Cardiovascular diseases remain a major global health issue and the leading cause of mortality among women [1]. Regarding presentation, management, and outcomes in cardiovascular diseases, it is crucial to further raise awareness of significant sex differences [2].

A potential sex-related difference is also of importance in atrial fibrillation (AF)/atrial flutter (AFL) [3]. As the most common arrhythmia, AF/AFL is associated with a substantial risk of stroke, heart failure, and other complications as well as increased mortality and represents a serious condition for those affected [4,5]. Especially in the case of highly symptomatic, multimorbid, and elderly patients, a rapid and safe acute treatment of AF/AFL is essential [6]. Emergency departments (EDs) are often the first point of contact for these patients within the health care system and, therefore, play a crucial role in further management. As initial management can affect the outcome, clinicians need to be aware of potential dissimilarities to ensure optimal individualised treatment [7].

Over the past years, knowledge about AF and acute management, especially rhythm-restoring or rate-controlling therapy, has significantly increased. Although there have been various advances in research regarding the optimal treatment regimen, a definite recommendation for one strategy over the other is lacking as none has proven to be superior. However, focussing on sex aspects, differences can be seen in various fields of AF/AFL, necessitating their distinct consideration in the overall management of AF/AFL [3]. These findings and consecutive needs were first highlighted by the European Society of Cardiology (ESC) guidelines on the management of AF published in 2016 [8].

Women tend to be older than men when they are first diagnosed. Regarding treatment strategies, there are inconsistent findings; however, a tendency can be seen that women receive primary rhythm control less often than men in acute therapy [9]. In addition, it has been reported that women are less likely to receive electrical cardioversion as a primary therapeutic approach [10]. When rhythm control is performed, women are usually older, have higher CHA_2_DS_2_-VASc scores, and more often have a history of stroke and arterial hypertension [11].

Moreover, in addition to acute management, the female sex itself has been identified to contribute to stroke risk in AF/AFL. However, studies show that oral anticoagulants are less frequently prescribed to women to prevent embolism, leading to potential harm from undertreatment [12].

Consecutively, sex-sensitive approaches in cardiovascular care have already made considerable progress. These include improvements in diagnostic accuracy, more tailored therapeutic interventions, and enhanced overall management strategies for women with AF/AFL [13]. However, as treatment strategies for AF/AFL continue to evolve, recommendations change accordingly and, therefore, it is crucial to emphasise sensitivity to sex differences to ensure equitable care for women and men [8,14,15]. By recognising, accounting for, and re-evaluating these sex-specific factors—such as differences in presentation, risk assessment, and treatment response—ED physicians can optimise decision-making processes to ensure equitable, effective, and safe emergency care for all patients. Therefore, the aim of the present analysis was to provide a long-term assessment of treatment standards at an arrhythmia centre, with a focus on sex-specific acute therapy.

## 2. Methods

### 2.1. Study Design, Setting, and Study Population

This analysis was conducted at the ED of the Medical University of Vienna, Austria, a 2200-bed tertiary care centre. The ED of the Medical University of Vienna, Austria, consists of an outpatient unit and associated critical care section, where approximately 90,000 patients are treated annually. Since 2012, over 4000 episodes of AF/AFL have been registered; AF/AFL visits per year range from 150 up to a maximum of 600 episodes per year. This analysis was part of a local prospective AF/AFL cohort study previously described in detail [16].

In brief, the underlying single-centre observational study was conducted using data from a prospective cohort documented in a registry maintained by the Department of Emergency Medicine, Medical University of Vienna, Austria. This registry included all consecutive patients aged 18 years and older who visited the ED for AF/AFL and provided written informed consent. Study nurses collected essential patient information, such as vital signs and pre-existing health conditions. Standard laboratory results were automatically extracted from the hospital’s electronic patient records. All treatment strategies at the ED were based on current local treatment standards prepared as standard operating procedures by consistently following the most recent European guidelines [8,14,17]. Any reoccurrence of sinus rhythm was detected by continuous monitoring and confirmed by ECG. The time of the reoccurrence of sinus rhythm was considered to be the time of the ECG-confirmed sinus rhythm.

The terms “sex” and “gender” are used in accordance with the Sex and Gender Equity in Research (SAGER) Guidelines. The SAGER Guidelines Checklist was used for this study [18]. Sex refers to the biological differences between males and females, including reproductive organs, chromosomes, and hormonal profiles. These biological characteristics are generally assigned at birth based on physical anatomy. Gender, on the other hand, encompasses the roles, behaviours, activities, expectations, and societal norms that cultures associate with being male or female. Gender identity is an individual’s personal sense of their own gender, which may or may not align with their biological sex.

In this study, the term “women” is used to refer to individuals with a female sex, which typically encompasses those assigned as female at birth based on biological characteristics. Conversely, the term “men” is employed to denote individuals with a male sex, generally referring to those assigned as male at birth. It is important to note that while these terms often align with biological sex, they also reflect broader social and cultural identities. The use of “women” and “men” in this context acknowledges both the biological aspects of sex and the gendered identities that individuals may embrace.

### 2.2. Statistics

Variables are presented as absolute values (*n*), relative frequencies (%), or as medians with 25th–75th interquartile ranges (IQRs). Group comparisons were performed using the Mann–Whitney U test for continuous variables and the chi-squared test or Fisher’s exact test for nominal variables. Kaplan–Meier failure functions were used to analyse time-to-event data. Furthermore, a proportional hazard regression was performed. The analysis of the main model was adjusted for the covariates of symptom severity, heart rate on admission, arterial hypertension, diabetes mellitus, COPD, coronary heart disease, previous stroke or transient ischemic attack, heart failure, valvular heart disease, rhythm on admission (AF/AFL), and previous attempts at cardioversion. The construction of this fully adjusted model was based on an assessment of verified influencing factors from a clinical perspective and proceeded stepwise to achieve the most parsimonious model. The effect of the variable ‘sex’ on time-to-event was assessed in the unadjusted model using the log-rank test statistic and, for the fully adjusted model, using the likelihood ratio test. After the comprehensive analysis of the entire cohort before and after 2016, clinically relevant subgroups were separately analysed to gain insights into the mechanisms underlying the observed effects. Therefore, cases were classified according to the primary treatment approach. The subgroup with spontaneous conversion was defined as cases in which sinus rhythm was achieved without any antiarrhythmic therapeutic measures, i.e., only interventions such as body positioning, fluid, and electrolyte replacement or anxiolysis.

Due to this categorisation being based on the primary treatment approach, transitioning from one group to another was not possible. The possibility of incorrectly analysing a single case in several groups was also prevented in this way. Competing risk due to mortality was assessed by analysing the mortality data available for all cases. As none of the patients analysed died during the observation period, it was ruled out. To control the false discovery rates in the context of multiple testing, we applied the Benjamini–Hochberg method. Model robustness was assessed using a sensitivity analysis, evaluating results from an unadjusted model, a fully adjusted model, and its variants with different observation periods (75th, 90th, and 99th percentiles of the time to restoration of sinus rhythm from the total sample). Where necessary, missing data were included as separate categories. Data analyses was conducted using Stata 18.0 (StataCorp., College Station, TX, USA). A two-sided *p*-value of < 0.05 was considered to be statistically significant.

## 3. Results

### 3.1. Baseline Characteristics

A total of 3661 cases were analysed. Of these, 2072 cases (56.6%) were recorded from the period 2012 to 2015 and 1589 cases (43.4%) were from the period 2016 to 2022. In total, 2040 cases (55.7%) were men. Atrial flutter was present in 688 (18.8%) cases. AF/AFL was diagnosed for the first time in 625 cases (17.1%).

The mean heart rate at admission was 131 bpm (IQR 112–147). Detailed information on baseline data regarding comorbidities, history of AF, and laboratory values at admission are shown in Table 1.

### 3.2. Treatment Approach and Outcomes

The frequency of spontaneous conversion to sinus rhythm in men and women in the total study population was 11.3% and 11.8% in the period from 2012 to 2015 and 11.4% and 12.7% in the period from 2016 to 2022. Although electrical cardioversion was the primary treatment approach in 37.8% and 41.1% of cases in men and women before 2016, the proportions reversed after 2016 to 43.5% and 35.5%, respectively. Among men, attempts at pharmacological cardioversion decreased from 32.3% before 2016 to 26.8% after 2016, while there was an increase among women from 25.0% to 28.1%. The use of rate control as a therapeutic approach was similar before and after 2016, both in terms of overall proportion and sex distribution. Although the rates of restored sinus rhythm in men and women were 70.8% and 71.2% before 2016, treatment success in women decreased after 2016, with corresponding relative frequencies of 71.8% and 68.6%. Table 2 provides details of the primary treatment approach and outcomes.

### 3.3. Time-to-Event Analysis

For the time-to-event analysis, 7015.86 h of time at risk was available in the analysis period from 2012 to 2015 and 5072.53 h of time at risk was available after 2016. The result of the log-rank test for analysing the influence of sex on the time to restoration of sinus rhythm in the unadjusted model showed no significance for cases in the period prior to 2016 (*p* = 0.810) but it was significant for cases in the period after 2016 (*p* = 0.015).

The fully adjusted model also showed a significant effect of sex on time to sinus rhythm restoration (likelihood ratio test, *p* = 0.013) for cases in the pre-2016 period but not for cases in the post-2016 period (*p* = 0.865).

**Figure 1:**
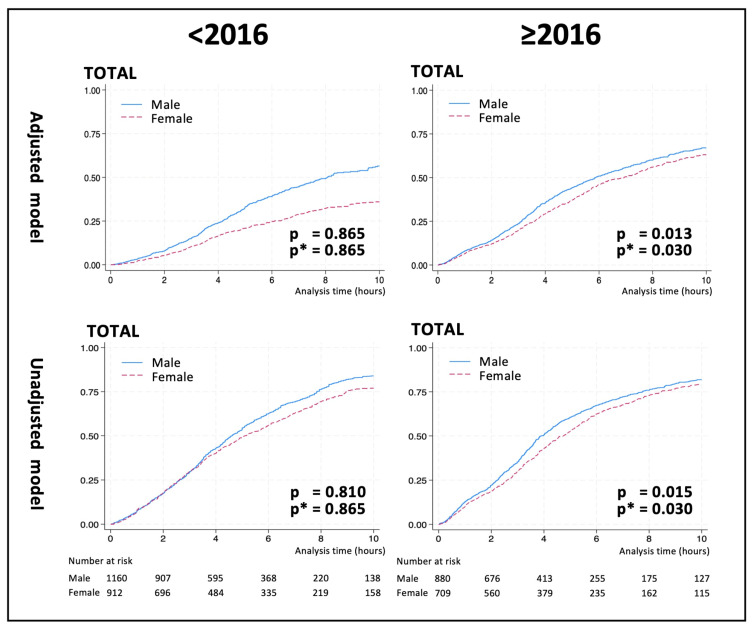
Kaplan–Meier failure functions of the adjusted and the unadjusted models for sinus rhythm restoration before and after 2016. * Benjamini–Hochberg-adjusted *p*-value. X axis: time (hours); y axis: relative numbers of successful treatment (%).

A subgroups analysis by primary treatment approach showed the same results for pharmacological rhythm control (likelihood ratio test on the fully adjusted model for cases before 2016, *p* = 0.193; after 2016, *p* = 0.035; see Supplementary Material). Further details of the results of the time-to-event analyses using the fully adjusted main model are shown in Figure 1.

### 3.4. Sensitivity Analysis

Similar effects were observed across the unadjusted model (before 2016, *p* = 0.810; after 2016, *p* = 0.015), the adjusted model (before 2016, *p* = 0.865; after 2016, *p* = 0.013), and the fully adjusted model variants with observation periods up to the 75th (6.12 h: before 2016, *p* = 0.733; after 2016, *p* = 0.035), 90th (8.86 h: before 2016, *p* = 0.894; after 2016, *p* = 0.035), and 99th (21.60 h: before 2016, *p* = 0.128; after 2016, *p* = 0.034) percentiles of the time to restoration of sinus rhythm for the total study population. These consistent findings support the robustness of our results.

## 4. Discussion

The present study uncovered a potentially critical concern in emergency medicine: the re-emergence of sex disparities in the treatment of acute AF in the ED. This study analysed a consecutive series of 3661 cases over an 11-year time period.

Sex differences in arrhythmias have been previously described. Although the same resources and treatment options are available, there are still differences in the care for men and women [19]. The 2016 ESC guidelines on AF first highlighted this potential equity gap in AF care [8]. For example, female sex was shown to have an influence on stroke risk in AF. Nevertheless, female patients seem to receive fewer oral anticoagulants than male patients, resulting in a potentially critical undertreatment [12]. In addition to stroke risk, treatment strategies also seem to differ between the sexes [20]. This was also true at our emergency department: we found a recurring sex gap in success rates and time to treatment success (restoration of sinus rhythm) after 2016. In particular, the primary strategy of pharmacological cardioversion appears to require critical reflection.

The literature shows that women tend to receive less electrical cardioversion but more pharmacological rhythm-restoring attempts than men. This was consistent with our findings: pharmacological cardioversion was used as a primary therapeutic strategy more often in women after 2016 (28.1%) than before 2016 (25.0%). The trend was reversed in men, with 32.3% before 2016 and 26.8% after 2016. Furthermore, prior to 2016, 37.8% of men and 41.1% of women were treated with electrical cardioversion as the primary approach but since 2016, the numbers have reversed (43.5% vs. 35.5%). However, success rates for women were higher than for men, both before 2016 (79.2% vs. 77.0%) and after 2016 (80.2% vs. 76.8%). These results suggest that, despite European-guideline-driven therapeutic standards at the study site, there has been a trend that reduces the use of electrical cardioversion and favours the use of pharmacological rhythm control in women. However, rhythm-restoring success rates suggest contradictory results as electrical cardioversion appears to be more successful in women than in men, while pharmacological rhythm control is less successful in women.

Another important difference was observed in time to treatment success. We found that after 2016, the restoration of sinus rhythm was significantly faster in men than in women. The median times to sinus rhythm in men and women were 2.7 versus 3.3 h for spontaneous conversion, 3.1 versus 3.6 h for electrical cardioversion, 3.8 versus 5.1 h for pharmacological cardioversion, and 5.1 versus 5.4 h for rhythm control. Likelihood ratio tests of the adjusted model for comparisons with and without sex as a factor were clearly significant for the overall study population (*p* = 0.013) and confirmed the recurring sex difference in the period after 2016. The analysis of patients with spontaneous conversion and primary rate control showed that the difference could not be explained by these treatment groups. The sex-specific difference was particularly observed in patients assigned to a primary strategy of pharmacological cardioversion. Here, the test for the effect of sex on achieving sinus rhythm was clearly significant for cases after 2016 (*p* = 0.035), while it was not significant for cases before 2016 (*p* = 0.193). The time to achieve sinus rhythm was significantly longer. The median time to achieve sinus rhythm before 2016 was 4.2 h for men and 4.1 h for women; it was 3.8 h for men and 5.1 h for women after 2016. Thus, over the course of the treatment era, there was not only a temporal reduction for men but also an increase for women. Furthermore, the difference in the median times to sinus rhythm between sexes was not only reversed in favour of men but also significantly increased.

The observed re-emergence of a sex gap has significant implications for the practice of emergency medicine. Due to the study design, an explanation for these findings can only be hypothesised. In our study cohort, the women were older than the men (before 2016: male 66 and female 72 years [median]; after 2016: male 63 and female 73 years [median]). In 2016, a potential difference in outcome regarding ED readmission for elderly patients was found in the literature [21]. Moreover, it was highlighted that female sex and older age were especially associated with complications and failure of electrical cardioversion [22]. Hence, this could align with the findings in our study, where women were treated with pharmacological methods more often and received electrical cardioversion as a primary strategy less often than men after 2016.

Furthermore, it has been found that women and men present different symptoms of AF/AFL, with women generally being more symptomatic than men [19]. Conversely, we found that the EHRA symptom score did not differ between sexes before and after 2016. A potential bias could lie within the perception of treating physicians because symptomatic women might not have been taken as seriously as their male counterparts, resulting in potentially less rapid and “aggressive” treatment for women.

A potential reason for the results of this study could lie in the allocation of treatment methods based on the patients’ AF history. For men, the frequency of pharmacological cardioversion decreased for those with a history of prior electrical cardioversion but remained stable for patients with a history of ablation. Conversely, women experienced an increased use of pharmacological cardioversion after reporting a history of ablation. This allocation pattern suggests different reactions to the surrogate information of more advanced disease compared with the previous era. This could potentially influence the efficacy of pharmacological treatments. The varying success rates and treatment assignments call for a critical and careful re-evaluation of current protocols and guidelines. Moreover, these findings underscore the importance of personalised treatment strategies that take into account not only the patient’s acute clinical presentation but also their AF history and broader health context.

Finally, a converse trend of the first highlighted equity in care in 2016 could have been observed in the findings of this analysis. As women and men have different needs and attributes, it is required to first recognise and understand these differences in order to achieve equality in healthcare. Instead of applying a uniform treatment approach for everyone, we should consider the specific epidemiology, pathophysiology, symptom burden, and prognosis of AF in men and women [14]. By identifying these differences, we can develop personalised treatment strategies tailored to the individual needs of women. This nuanced approach could not only promote equality but also enhances the overall effectiveness of medical interventions.

From the authors’ perspective, there are several key elements that must be considered to ensure future equity of care in AF. First of all, a thorough comprehensive assessment of the patient’s history of AF and general health should be an integral part of the treatment (shared) decision-making process. This approach could help to identify patients who could benefit most from certain interventions and thus optimise outcomes [23]. Secondly, emergency medical staff should be trained to recognise and mitigate implicit biases that may influence treatment decisions. Awareness programmes highlighting the re-emergence of the sex gap and its impact could promote a more equitable approach to emergency care [24]. Additionally, treatment allocation should be reviewed to ensure that decisions are not unduly influenced by sex bias. The introduction of more robust decision-support systems in EDs could help to make more objective treatment decisions, potentially reducing the sex gap [25]. Furthermore, continuous research is essential to monitor the effectiveness of the changes implemented and to understand the evolving dynamics of sex inequalities in emergency medical treatment [24]. Finally, pharmacological cardioversion protocols should be revised. The decline in success rates for women suggests a need to re-examine the criteria and conditions under which pharmacological cardioversion is chosen. It may be beneficial to develop sex-specific guidelines or tailored protocols that address the unique progression patterns and treatment responses in women [19].

## 5. Strengths and Limitations

A key strength of this study was its real-world setting, which supported robust findings that could be generalised beyond the selected study population. The study was conducted at the ED of the Medical University of Vienna, which has both an outpatient clinic and an intensive care unit, and thus benefits from the necessary infrastructure to immediately initiate cardioversion procedures. This setting ensures immediate access for patients from diverse socio-cultural backgrounds as it is a publicly accessible facility. Following rigorous guideline-driven treatment protocols, hemodynamically unstable patients receive immediate electrical cardioversion. When patients’ conditions allow, treatment decisions are based on comprehensive initial evaluations, including echocardiography and laboratory results, which are quickly available due to the local centralised laboratory. This tertiary care infrastructure supports the high standard of care and enables the standardised application of international guidelines. Consequently, this setup facilitated the management of a real-world cohort using maximum clinical resources and documentation by specialised personnel.

Several limitations must be acknowledged. First, the single-centre design restricted the generalisability of our results as patient characteristics, treatment protocols, and healthcare infrastructures may differ across institutions and regions. Second, the observational nature of the study limited the ability to establish causal relationships between variables as unknown confounding factors may have influenced the observed associations. Although we observed sex differences in the treatment success and timing of sinus rhythm restoration, the underlying causes could not be inferred from our results. As an observational study, our analysis was limited to associations and not causal inferences. Potential contributing factors, such as biological differences, comorbidities, variations in treatment response, or sex-related inequalities in healthcare delivery, require further investigation in controlled, hypothesis-driven studies. Future research, ideally with larger, multicentre study designs, is needed to validate our findings. Further, due to subgroups being classified according to the primary treatment approach, the resulting subgroup-specific success rates should not be interpreted as those of a single method alone but rather as a combination of methods applied during the clinical course. However, these rates remained accurate for the respective primary treatment approach undertaken. Additionally, the potential impact of missing data is a notable limitation. Although we avoided strict assumptions about missing data by incorporating them as separate categories within the models when necessary, it is essential to recognise that this approach may not have entirely eliminated bias.

Finally, it is important to note that we did not include a formal interaction analysis to assess if the effect of sex on time to sinus rhythm restoration and treatment success changed over time. Instead, we used time-to-event analysis and proportional hazard regression models, stratifying cases into pre- and post-2016 groups to examine sex-specific differences within each period. Although this allowed for a temporal comparison, a formal interaction analysis could probably provide a more rigorous test of sex-related changes over time.

## 6. Conclusions

The re-emergence of a sex gap in the success rates and time to sinus rhythm restoration in patients with acute AF is concerning and needs re-evaluations of treatment protocols, enhanced decision-making processes, and continued research to ensure equitable, effective, and safe emergency care for all patients.

## Figures and Tables

**Table 1:** Baseline data regarding comorbidities, AF history, and laboratory values at admission of the total study population.

	<2016		≥2016	
	Male	Female	Male	Female
	*n* = 1160	*n * = 912	*n * = 880	*n * = 709
Age, years (IQR)	66 (55–73)	72 (65–79)	63 (52–73)	73 (64–78)
**Comorbidities**				
Heart failure, *n* (%)	283 (24.4)	230 (25.2)	91 (10.3)	75 (10.6)
Valvular disease, *n* (%)	289 (24.9)	251 (27.5)	183 (20.8)	208 (29.3)
Hypertension, *n* (%)	730 (62.9)	637 (69.8)	487 (55.3)	450 (63.5)
Diabetes mellitus, *n* (%)	177 (15.3)	146 (16.0)	121 (13.8)	104 (14.7)
Previous stroke, *n* (%)	77 (6.6)	67 (7.3)	55 (6.3)	76 (10.7)
CAD, *n* (%)	253 (21.8)	119 (13.0)	172 (19.5)	78 (11.0)
COPD, *n* (%)	108 (9.3)	94 (10.3)	77 (8.8)	46 (6.5)
** AF history **				
Atrial flutter, *n* (%)	216 (18.6)	145 (15.9)	196 (22.3)	131 (18.5)
Heart rate (admission), bpm (IQR)	127 (107–145)	130 (111–146)	132 (113–149)	133 (117–148)
First AF, *n* (%)	185 (15.9)	127 (13.9)	174 (19.8)	139 (19.6)
EHRA, median (IQR)	2 (0–2)	2 (0–2)	2 (2–3)	2 (2–3)
CHA2DS2-VASc (IQR)	2 (1–3)	3 (2–4)	2 (1–3)	3 (2–5)
Previous ryhthm control, *n* (%)	438 (37.8)	293 (32.1)	398 (45.2)	292 (41.2)
Previous Ablation, *n* (%)	154 (13.3)	163 (17.9)	163 (18.5)	137 (19.3)
** Laboratory **				
Haematocrit, % (IQR)	42 (38–45)	40 (37–44)	41 (38–45)	42 (38–45)
WBC, G/I (IQR)	8 (7–10)	8 (7–10)	8 (7–10)	8 (7–10)
Creatinine, mg/dL (IQR)	1.0 (0.9–1.2)	1.0 (0.8–1.2)	1.0 (0.8–1.3)	1.0 (0.9–1.2)
NT-proBNP, pg/mL (IQR)	1108 (345–2753)	1128 (474–2816)	1101 (418–2487)	1118 (384–2765)
hs-Troponin T, ng/L (IQR)	15 (9–28)	14 (8–26)	14 (8–23)	15 (9–27)
CRP, mg/dL (IQR)	0.4 (0.2–1.2)	0.3 (0.1–0.9)	0.4 (0.2–1.0)	0.4 (0.1–1.2)
INR (IQR)	1.1 (1.0–1.6)	1.5 (1.1–2.7)	2.3 (1.3–3.1)	1.2 (1.0–2.2)

**Table 2:** Therapy strategy and restoration rates of sinus rhythm in the total study population.

	<2016		≥2016	
	Male	Female	Male	Female
	*n* = 1160	*n* = 912	*n* = 880	*n* = 709
Spontaneus conversion				
Sinus rhythm, *n* (%)	131 (11.3)	108 (11.8)	100 (11.4)	90 (12.7)
Time to sinus rhythm, h (IQR)	2.7 (1.4–5.5)	2.8 (1.3–5.6)	2.7 (1.1–5.0)	3.3 (1.8–5.5)
Electrical cardioversion				
Attempts, *n* (%)	439 (37.8)	375 (41.1)	383 (43.5)	252 (35.5)
Sinus rhythm, *n* (%)	338 (77.0)	297 (79.2)	294 (76.8)	202 (80.2)
Time to sinus rhythm, h (IQR)	3.5 (2.3–5.9)	3.6 (2.2–5.7)	3.1 (1.8–4.6)	3.6 (1.7–5.2)
Pharmacological cardioversion				
Attempts, *n* (%)	375 (32.3)	228 (25.0)	236 (26.8)	199 (28.1)
Sinus rhythm, *n* (%)	211 (56.3)	108 (47.4)	131 (55.5)	98 (49.2)
Time to sinus rhythm, h (IQR)	4.2 (2.6–6.3)	4.1 (2.1–6.6)	3.8 (2.5–6.6)	5.1 (2.8–7.1)
Rate control				
Attempts, *n* (%)	215 (18.5)	201 (22.0)	161 (18.3)	168 (23.7)
Sinus rhythm, *n* (%)	141 (65.6)	136 (67.7)	107 (66.5)	96 (57.1)
Time to sinus rhythm, h (IQR)	4.9 (2.8–7.3)	5.0 (2.5–7.7)	5.1 (2.5–9.1)	5.4 (3.2–9.7)
Total				
Sinus rhythm, *n* (%)	821 (70.8)	649 (71.2)	632 (71.8)	486 (68.6)

## Data Availability

The original contributions presented in this study are included in the article. Further inquiries can be directed to the corresponding author.

## Supplementary Materials

The following supporting information can be downloaded at: https://www.mdpi.com/article/10.3390/jcm14041250/s1, Figure S1. Kaplan Meier failure functions for sinus rhythm restoration under different therapeutic approaches before and since 2016. X axis: time (hours); y axis: relative numbers of successful treatment (%).
