# Supplementary material for: Mind the Gap: Recurrence of Sex-Related Differences in Patients with Acute Atrial Fibrillation in the Emergency Department—A Retrospective Cohort Study
Source: J Clin Med. 2025 Feb 13;14(4):1250. doi: 10.3390/jcm14041250 (PMC11856482; doi:10.3390/jcm14041250)
Supplement: Supplementary file 1 [file jcm-14-01250-s001.zip › jcm-3390039-supplementary.pdf]

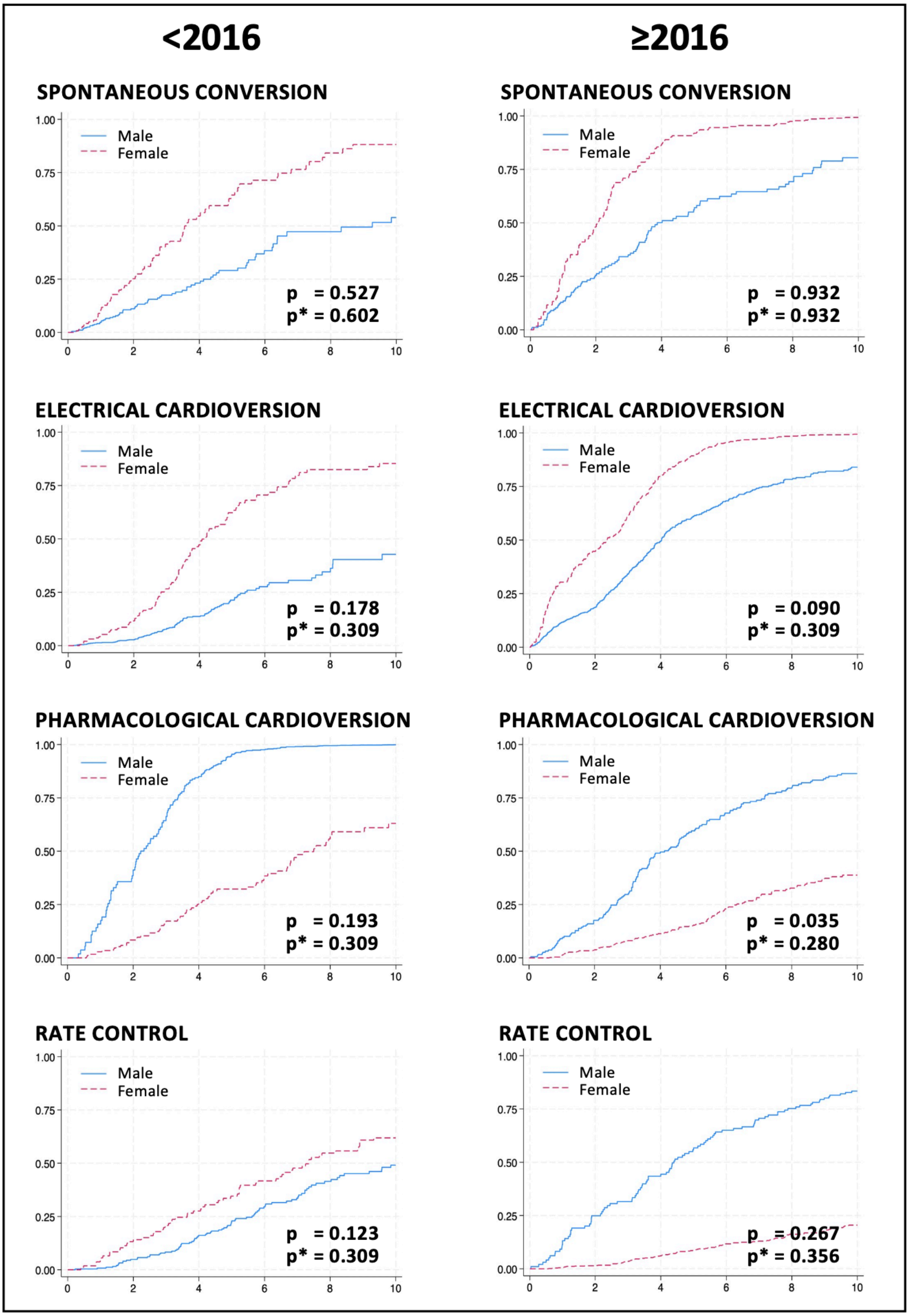

Supplementary figure S1 Kaplan Meier failure functions for sinus rhythm restoration under different therapeutic approaches before and since 2016. X axis: time (hours); y axis: relative numbers of successful treatment (%).
